# Combined Heart-Kidney Transplantation: Indications, Outcomes, and Controversies

**DOI:** 10.14797/mdcvj.1139

**Published:** 2022-09-06

**Authors:** Syed Adeel Ahsan, Ashrith Guha, Juan Gonzalez, Arvind Bhimaraj

**Affiliations:** 1 Methodist DeBakey Heart & Vascular Center, Houston Methodist, Houston, Texas, US; 2 The Kidney Institute, Houston Methodist, Houston, Texas, US

**Keywords:** orthotopic heart transplantation, deceased donor kidney transplantation, chronic kidney disease, heart-kidney transplantation, transplant

## Abstract

Renal dysfunction, a prevalent comorbidity in advanced heart failure, is associated with significant morbidity and mortality after heart transplantation. In the recent era, the field of combined heart-kidney transplantation has experienced great success in the treatment of both renal and cardiac dysfunction in end-stage disease states, and the number of transplants has increased dramatically. In this review, we discuss appropriate indications and selection criteria, overall and organ-specific outcomes, and future perspectives in the field of combined heart-kidney transplantation.

## Introduction

Heart transplantation is the definitive therapy for end-stage heart failure, and significant advances in surgical technique and medical management have resulted in improved survival post heart transplant (HTx). Renal dysfunction is highly prevalent in patients with advanced heart failure and can be due to reversible cardiorenal syndrome, independent advanced kidney disease, or a combination of both. Renal dysfunction is also a common post-transplant morbidity and a significant contributor to long-term mortality.^1^ Because many post-transplant factors can contribute to renal damage, it is important to carefully assess the risk of early renal dysfunction and the possible need for earlier-than-necessary dialysis.

Combined heart-kidney transplantation (HKTx), first described in 1978 by Norman et al.,^2^ has become a recognized therapy for simultaneous end-stage cardiac and renal dysfunction. Consideration for HKTx in patients with heart failure and irreversible renal dysfunction or chronic kidney disease (CKD) is supported by United Network for Organ Sharing (UNOS) registry analysis, which shows similar survival for HTx compared with HKTx,^3^ increased mortality associated with lower pre-HTx estimated glomerular filtration rate (eGFR),^4^ and improved post-transplant survival with HKTx versus HTx in patients with eGFR < 37 mL/min/1.73m^2^.^5^ This has resulted in a dramatic rise in HKTx in the United States (US), with a nearly 5-fold increase from 2011 to 2021.^6^ At the same time, however, UNOS data comparing HKTx to kidney transplant after HTx shows a significant survival disadvantage for simultaneous multiorgan transplant as opposed to sequential. This suggests that some patients may be better served by deciding to have a renal transplant based on their renal function after HTx.^7^ As organ demand continues to outstrip supply, the decision to allocate two organs simultaneously to a single recipient requires careful patient selection and management to ensure the best possible outcomes.^8^ In this review, we discuss appropriate recipient selection, overall and organ-specific outcomes, and future directions for heart-kidney transplantation.

## Indications

The decision to consider HKTx is driven by poor outcomes related to renal failure after HTx. Given the possibility of renal recovery after transplant in the case of cardiorenal syndrome without intrinsic renal disease, initial thought was given to determining which patients had irreversible renal dysfunction that would preclude HTx.^9^ The 24th Bethesda Conference in 1993 described a creatinine clearance of < 50 mL/min or serum creatinine > 2 mg/dL as an absolute contraindication to HTx.^10^ A majority of US centers in 1996 believed a serum creatinine > 3 mg/dL was a relative contraindication to transplantation,^11^ and in 2006 the International Society of Heart Lung Transplantation (ISHLT) recommended using a pre-transplant eGFR < 40 mL/min to describe irreversible renal dysfunction as a relative contraindication to HTx along with supporting studies such as renal ultrasonography, proteinuria, and renal arterial disease evaluation.^12^ This level was reduced to < 30 mL/min/1.73m^2^ in the 2016 ISHLT guideline update.^13^ Given that UNOS registry data consistently suggests a survival benefit to HKTx over HTx in patients requiring pre-transplant dialysis, it is clear that patients with dialysis dependence would benefit from HKTx.^3,14^ However, renal dysfunction (eGFR < 60 mL/min/1.73m^2^) without dialysis dependence is associated with worse outcomes post HTx, including increased mortality, end-stage renal disease (ESRD), and need for kidney transplant.^4^ Therefore, patients at less extremes of renal dysfunction may receive additional benefit with multiorgan transplant.

Mayo Clinic analysis of 12 patients found that HKTx in patients with an eGFR < 40 mL/min/1.73m^2^ was associated with good survival and a lower incidence of rejection and coronary allograft vasculopathy (CAV).^15^ Similarly, analysis of Scientific Registry of Transplant Recipients data over almost 20 years suggested a survival benefit for HKTx in patients with eGFR < 40 mL/min/1.73m^2^ or dialysis dependence as compared to 40 to 60 mL/min/1.73m^2^.^16^ UNOS data analysis of 593 patients with HKTx found that when stratified into quintiles of renal function, patients in the lowest quintile of renal function undergoing HTx had decreased survival compared to all others, and it suggested an eGFR < 37 mL/min/1.73m^2^ as the cutoff for HKTx to improve post-transplant survival to that of HTx patients without renal dysfunction.^5^ Agarwal et al. directly compared UNOS registry data of patients with renal dysfunction undergoing HKTx to those with HTx and found a survival benefit for HKTx in patients with eGFR < 45 mL/min/1.73m^2^ but no difference in patients with eGFR > 45 mL/min/1.73m^2^.^17^ Therefore, at a 2019 consensus conference held in Boston, experts in cardiothoracic and kidney transplantation recommended that heart transplant candidates with an established eGFR < 30 mL/min/1.73m^2^ be considered for HKTx, and that patients with an eGFR of 30 to 44 mL/min/1.73m^2^ with evidence of intrinsic CKD, such as proteinuria or decreased kidney size, also be considered on an individual basis. They also stated that patients with an established eGFR above 45 mL/min/1.73m^2^ were unlikely to be appropriate candidates for HKTx.^18^

These recommendations do not apply to patients with rare disorders, such as those with a solitary kidney or those with proteinuria unrelated to dysfunction in glomerular filtration. In patients with either a congenital or acquired solitary kidney, eGFR calculation by creatinine or cystatin-c measurement is unreliable and may necessitate use of measured GFR.^19,20,21,22^ In disease states such as end-stage cardiac amyloidosis, renal manifestations may be limited to proteinuria without significant impact on markers of GFR.^23^ Determining the need for kidney transplantation in these patients requires additional expertise and decision making that is outside the scope of this paper.

**Figure 1:**
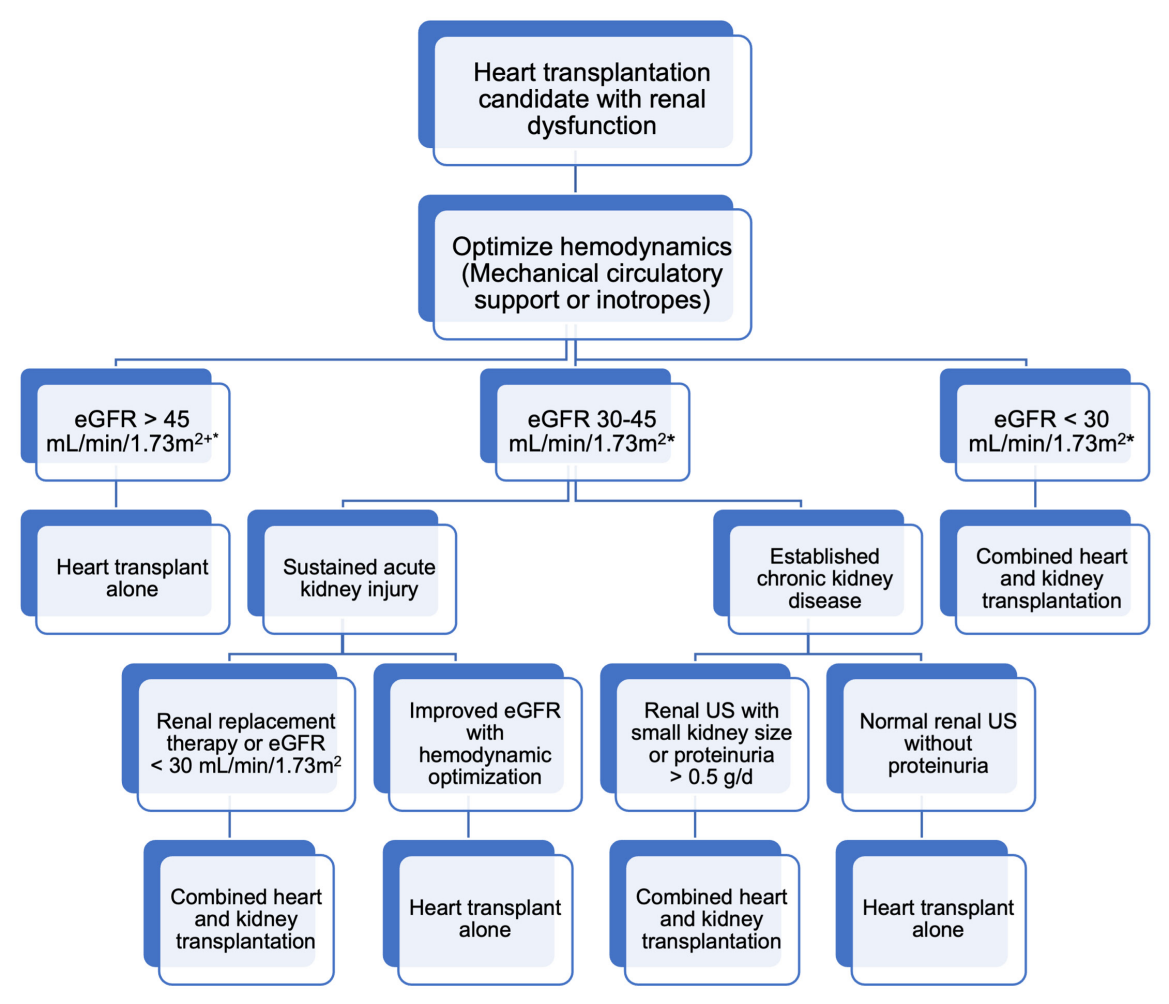
Algorithm for the selection of patients requiring heart transplantation who would benefit from combined heart-kidney transplantation. eGFR: estimated glomerular filtration rate; US: ultrasound. ^+^In the absence of significant structural abnormality (uninephric state) or proteinuria *eGFR as measured on at least two separate occasions.

Selection criteria for HKTx recipients should reflect the previously mentioned nuances in outcomes with renal function and also allow for incorporation of both renal structural disease and function in determining the need for HTx versus HKTx (Figure 1).

## Outcomes

### Overall survival

From the first analysis of UNOS outcomes data published by Narula et al., HKTx has yielded at least similar intermediate survival rates as HTx, with signals towards less cardiac rejection.^24^ This is supported by multiple single-center studies showing that HKTx over different eras of transplantation techniques and immunosuppression has resulted in at least equivalent or acceptable survival compared with HTx.^15,25,26^ More recent analysis of UNOS data has attempted to utilize complex statistical methodology to compare similar groups of HKTx and HTx patients to identify whether a survival advantage may be offered by HKTx. Agarwal et al. found comparable long-term survival between all HKTx and HTx patients in UNOS up to 2019 (median 12.4 vs 11.3 years, *P* = .053), with significant survival benefit in patients with eGFR < 45 mL/min/1.73 m^2^ (median 13 vs 10.2 years; *P* < .001), and patients on pre-transplant dialysis (median 12.4 vs 9.9 years; *P* < .01).^17^ While UNOS data suggests age greater than 65 years may be associated with worse survival in HKTx, single-center data from Cedars-Sinai hospital suggests that survival, along with ICU stay, appears to be similar between HKTx and HTx in patients over 65 years.^27,28^

### Cardiac Outcomes

The survival benefit after HKTx appears to be driven by a significant increase in cardiac graft survival. UNOS data analysis shows that while 1-year survival is similar, 5- and 10-year cardiac graft survival is significantly better in HKTx compared with HTx patients.^17^ Multiple single-center studies show significantly lower rates of cardiac allograft rejection in HKTx compared with HTx.^15,25,29^ Rates of acute cardiac rejection within the first year and at 5 and 10 years is lower in HKTx compared with HTx patients. Freedom from cardiac allograft rejection events is significantly better in HKTx compared with HTx patients, with a much longer time to first rejection episode, and HKTx patients ultimately undergo treatment for rejection at half the rate of HTx patients.^17,30^ Multiple possible etiologies have been suggested, including a much higher antigenic load with dual solid organs resulting in “immune paralysis”—namely, induction of partial tolerance leading to fewer rejection events.^15^ In fact, relative antigen levels on kidneys were found to be 3-fold greater for Human Leukocyte Antigen (HLA)-ABC, and 90-fold greater for HLA-DR than those on heart tissue.^31^ Preliminary animal model studies also suggest that the donor kidney possesses cells that migrate to the host thymus and induce tolerance to donor antigen as well.^15,32^ Multiple single-center studies have also shown decreased rates of angiographic CAV in patients with HKTx, with more recent data using high-resolution intravascular ultrasound (IVUS) imaging showing decreased progression of the coronary artery wall thickness in patients with HKTx compared to HTx. This difference persisted even when patients with HKTx were matched to those with HTx with similar CAV risk profiles and rejection burden, while also accounting for confounding factors such as induction with anti-thymocyte globulin and immunosuppression with mTOR inhibitors, both of which are associated with lower rates of CAV.^15,24,25,33,34,35^ Therefore, the immunologic attenuation induced by concurrent kidney transplantation could possibly be responsible for not only reduced acute rejection episodes but also for decreased CAV rates and therefore lead to improved cardiac allograft survival in HKTx patients.

### Renal Outcomes

Renal outcomes after HKTx should be considered in comparison to HTx and for patients undergoing kidney transplant alone (KTx). While cardiac allograft survival benefits from HKTx in appropriate patients, it is imperative to keep in mind that kidneys are allocated to multiorgan transplant recipients ahead of those requiring KTx. Also, patients receiving multiorgan transplants are generally allocated higher-quality kidneys than patients undergoing KTx. The Kidney Allocation System (KAS) as designed by UNOS was recently restructured to emphasize longevity matching; namely, matching recipient post-transplant survival to allograft survival. However, in redirecting better-quality organs to recipients with shorter life expectancies, HKTx may lead to decreased renal allograft lifespan. Therefore, allocating a kidney to a patient with only moderate renal insufficiency may result in less survival benefit overall than if it were given to a patient listed for KTx.^35,36,37^

Reduced eGFR prior to HTx is associated with increased ESRD and need for dialysis post-transplant. Given the significantly increased mortality risk conferred by the need for persistent dialysis post HTx, impaired renal function prior to transplant is associated with decreased survival persisting up to 10 years post-HTx.^4,38^ As discussed previously, HKTx ameliorates this survival impairment, particularly in patients with worse renal function or dialysis dependency. HKTx is also associated with a decreased need for permanent dialysis and repeat kidney transplantation in multiple single-center studies.^15,39^ Overall HKTx is associated with improved renal and survival outcomes compared with HTx in patients with impaired renal function.

When comparing overall survival in separate populations, HKTx patients have increased in-hospital mortality, resulting in reduced 1-year survival rates compared with all KTx patients. While this reflects the complexity, increased comorbidities, and hemodynamic impairment associated with multiorgan transplant, particularly thoracic compared with abdominal, it remains a cause of concern when allocating renal allografts away from patients awaiting KTx.^40^ UNOS data supports this, showing lower rates of graft survival both in the first year post-HKTx as opposed to KTx (84.5% compared to 89.8%, *P* < .001) and higher relative risk of graft loss for HKTx (HR 1.41; 95% CI, 1.13-1.76). These rates were driven by increased mortality rates in HKTx patients.^14^ Multiple single-center studies, however, do not show a significant difference in renal graft survival among matched populations of HKTx and KTx, although certain risk factors associated with higher graft failure rates were identified. These seemed to be centered around hemodynamic instability and prolonged operating time, such as prior cardiac surgery, prior heart or kidney transplant, and requirement for extracorporeal membrane oxygenation support.^40,41,42^ Overall, this suggests that utilization of kidney allografts for HKTx patients is appropriate, with care taken to identify patients at increased risk of poor outcomes.

## Future Perspectives

While HKTx is increasing significantly across the US, seemingly with excellent outcomes and better profiling of which patients are likely to benefit from graft allocation, several ethical and pragmatic issues need to be addressed. One major concern is the fact that no national policy exists for multiorgan transplantation. This was addressed by an Organ Procurement and Transplantation (OPTN)/UNOS Ethics Committee in a 2019 white paper, which states that the OPTN Final Rule requires development of allocation policies “specific for each organ type or combination types to be transplanted into a transplant candidate.”^43^ The lack of such a policy can create inequity in the organ distribution process, both in rate and time to transplantation. This highlights the need for a nationwide policy directed towards identifying those patients who can most benefit from HKTx and ensuring they receive appropriate allocation while not putting patients with possible marginal need at a substantial disadvantage. For example, while some patients with marginal renal function (ie, eGFR 30-44 mL/min/1.73m^2^) have worse outcomes after HTx, others may have renal recovery; however the clinical characteristics that differentiate these patients are not described in current guidelines. Therefore, while ethical concerns exist regarding allocating two organs to a single individual, denying these marginal candidates kidney allocation may impact their survival and cause harm not only at an individual level but also on the population level given the potential loss of the cardiac allograft.^18^

One possible means of improving post-HTx survival in patients who develop renal dysfunction is consideration of kidney transplant after HTx. Both single-center and UNOS registry data suggest that patients undergoing renal transplant after prior HTx have similar survival to HTx patients.^44,45^ In recent analysis comparing HKTx with KTx after HTx, HKTx had a 4-fold higher risk of death, even after multivariable analysis accounting for multiple risk factors and transplant center expertise. Despite that, KTx after HTx rates continue to drop across the US, corresponding to the increase in HKTx rates.^7^ Interestingly, despite concern that delayed renal transplant could contribute to early post-HTx mortality, UNOS data suggests that immediate (30-day) post-transplant dialysis rates are higher in HKTx patients than HTx, even in those patients with the lowest quintiles of renal function.^5^ This suggests that delayed KTx after HTx would not necessarily impact immediate post-HTx survival.

These issues are not unique to the HKTx population, and similar concerns led to development of a UNOS Liver-Kidney transplant (LKTx) policy implemented in 2017. The major components of this policy include formally describing eligibility criteria for LKTx that identify the likelihood of irreversible renal dysfunction (namely presence of CKD, sustained acute kidney injury, or metabolic disease) and describing the creation of a “safety net.” This safety net was developed to alleviate the same concerns leveled for HKTx: that development of stringent criteria for LKTx would lead to some potential candidates “falling through the cracks,” resulting in impaired outcomes due to ongoing renal dysfunction. The safety net provides kidney match classification priority to all liver recipients meeting renal dysfunction criteria between 2 and 12 months after liver transplant.^46^ Recent UNOS analysis appeared to show favorable results of this policy, and while there was a reduction in LKTx, it was offset by a corresponding increase in KTx after liver transplant, with minimal impact in survival outcomes.^47^

Development and implementation of such standardized policies was therefore recommended at the Boston consensus conference on HKTx along with the description of a safety net policy. While this policy might impact HKTx rates, it would not necessarily impact early HTx outcomes. It also may have the advantages of avoiding kidney allocation to patients who would recover renal function after HTx and avoiding heart transplant-associated perioperative and early postoperative hemodynamic instability that could contribute to renal graft dysfunction. This would ultimately allow for judicious use of donated kidneys without causing harm to HTx patients.

## Conclusion

Combined heart and kidney transplantation is a well-established therapy for patients with end-stage heart failure and renal dysfunction. While the number of HKTx procedures is growing rapidly, the selection criteria to identify the most appropriate candidates continue to be refined. Despite this, outcomes of HKTx are highly favorable, with comparable if not improved patient survival, improved cardiac graft survival, decreased rates of rejection and CAV, and reasonable renal outcomes compared with HTx. Given continued organ scarcity, further efforts must be made to provide multiple organs to those who truly need them without harming marginal candidates. Therefore, focus should be directed towards implementing a unified policy for HKTx with the likely incorporation of a “safety net” policy.

## Key Points

- Simultaneous heart-kidney transplantation (HKTx) is an established therapy for end-stage heart and renal failure.
- HKTx is indicated for patients with heart failure who are either dialysis dependent or have persistent irreversible renal dysfunction.
- Combined HKTx outcomes are highly favorable, with excellent overall and allograft survival.
- Development of a standardized HKTx policy is essential to continued outcome improvement and appropriate allocation of multiple organs.
